# Serological survey of *Toxoplasma gondii * in Tibetan mastiffs (*Canis lupus familiaris*) and yaks (*Bos grunniens*) in Qinghai, China

**DOI:** 10.1186/1756-3305-5-35

**Published:** 2012-02-13

**Authors:** Meng Wang, Yan-hua Wang, Qiang Ye, Peng Meng, Hong Yin, De-lin Zhang

**Affiliations:** 1 Lanzhou Veterinary Institute, Chinese Academy of Agriculture Sciences, 1 Xujiaping, Chengguan, Lanzhou, Gansu, China

**Keywords:** *Toxoplasma gondii *, Survey, Tibetan mastiff, Yak, IHAT

## Abstract

**Background:**

*Toxoplasma gondii * is an amphixenosis which has extensive hosts. In recent years, the prevalence of *T. gondii * in China has been reported, while little is known on the survey of *T. gondii * infection in northwest China, especially in yaks (*Bos grunniens*) and Tibetan mastiffs (*Canis lupus familiaris*). The current study survey the infection of *T. gondii * in Tibetan mastiffs and yaks in Qinghai Province, China.

**Methods:**

The indirect hemagglutination test (IHAT) was used to examine *T. gondii * antibodies in 1 795 serums, including 192 Tibetan mastiffs and 1603 yaks in Qinghai Province, China.

**Results:**

In this study, the seroprevalence of *T. gondii * infection was 8.52%. Twenty (10.42%) of 192 serums of Tibetan mastiffs and 133 (8.30%) of 1603 serums of yaks were seropositive. The seroprevalence of *T.gondii * infection in Tibetan mastiffs in breeding farm (1.08%) was lower than that in the field (19.19%), and the difference was statistically significant (*P* <0.05). The seroprevalence of antibodies to *T.gondii * in yaks ranged from 5.45% to 13.28% among the four different areas. The seroprevalence in different age groups were determined with apparent association.

**Conclusions:**

The results indicated that *T.gondii * infection was prevalent in Tibetan mastiffs and yaks, which have implications for public health in this region. To our knowledge, this is the first seroprevalence survey of Tibetan mastiffs infected by *T. gondii * in The People’s Republic of China.

## Background

*Toxoplasma gondii *, an obligate intracellular protozoan parasite, is one of the common parasitic infections of man and other warm-blooded animals [1-3], which may cause fetal diseases with severe problems, such as abortion, encephalitis, mental retardation and blindness in congenital cases [2]*.* There are two main routes of infection of *T.gondii *: through the ingestion of uncooked and raw infected meat, and through the ingestion of the oocysts present in the environment, from the faeces of infected cats [1,4].

Recently, the seroprevalence of *T. gondii * infection in yaks (*Bos grunniens*) have been reported in Qinghai Province, northeast of Tibetan Plateau [5,6], while little is known about the infection of *T. gondii * in Tibetan mastiffs (*Canis lupus familiaris*), living in high altitude and cold plateaus (over 3000 m above sea level). Tibetan mastiff is a canine species, known as the oldest and most ferocious dog in the world and has been introduced and domesticated into many other countries as pet.

The objectives of the present study were to investigate seroprevalence of *T. gondii * infection in yaks, as well as in Tibetan mastiffs from Qinghai Province, northwestern China. The control toxoplasmosis of yaks and Tibetan mastiffs reared on farms should be paid more attention to in China.

## Methods

1. The sites investigatedThe present study was carried out in Qinghai Province (31°-39°N, 88°-103°E), People’s Republic of China, which lies on the northeastern Tibetan Plateau. Average elevation of the survey province is 3 000 m above sea level, which extends about 724 000 km^2^. The annual precipitation is below 400 mm and average annual temperature is between 5.7°C and 8.5°C, and difference of daily temperature is large. The survey regions are typical continental climate-altitude.

2. Serum samplesBlood samples were collected by local veterinary practitioners from 1 603 yaks and 192 Tibetan mastiffs between 2009 and 2010. All the operations were humane according to the animal welfare. The serum samples of Tibetan mastiffs were collected from four breeding farms and fields in counties of Yushu and Wulan. The numbers of samples from each region are shown in Table 1. The serum samples of yaks were collected from Yushu (271), Wulan (359), Dulan (478) and Guinan (495) (Table 2). Each blood sample was then centrifuged (1 000 rpm) and sera were stored at −20 °C.

3. Serological testAccording to the agricultural industry standards, People’s Republic of China NY/T 573–2002, a commercial indirect hemagglutination test (IHAT) kit (Lanzhou Veterinary Institute, Chinese Academy of Agriculture Sciences) was used to detect antibodies to *T. gondii *. According to the manufacturer’s instructions, a 96 well plate with V well bottom was used at the screening dilutions of 1:4, 1:16, 1:64, 1:256, 1:1 024 and 1:4 096. Samples reacting above 1:64 were considered positive for *T. gondii * antibodies.

4. Statistical analysisThe differences in the prevalence rates of *T.gondii * between regions and age groups in Tibetan mastiffs and yaks were analyzed by Chi-square analysis in SPSS (Statistical Analysis System, Version 13.0). Values of *P* <0.05 were considered as statistically significant.

**Table 1:** Prevalence of *T. gondii * infection in Tibetan mastiffs by areas and antibody titers in Qinghai Province

**Origins**		**NO. examined**	**IHA**					**Positive**	
			**≤1:16**	**1:64**	**1:256**	**1:1024**	**1:4096**	**NO.**	**%**
Breeding Farm	A	50	49	1	0	0	0	1	2.00
	B	11	11	0	0	0	0	0	0
	C	12	12	0	0	0	0	0	0
	D	20	20	0	0	0	0	0	0
Field	Yushu	30	24	2	1	2	1	6	20.00
	Wulan	69	56	5	3	4	1	13	18.84
**Total**		192	172	8	4	6	2	20	10.42

**Table 2:** **Prevalence of *T. gondii *infection in yaks by areas and gender in Qinghai Province**

**Biometric data**	**Gender**		**Total**	**Areas**			
	**Male**	**Female**		**Yushu**	**Wulan**	**Dulan**	**Guinan**
NO. examined	763	840	1603	271	359	478	495
NO. positive	61	72	133	36	44	26	27
Prevalence (%)	7.99	8.57	8.30	13.28	12.26	5.44	5.45

## Results

A total of 153 (8.52%) of 1 795 serum samples from Tibetan mastiffs and yaks were positive by IHAT. Of these, 10.42% of Tibetan mastiffs were seropositive and antibodies titers were 1:4096 in 2, 1:1024 in 6, 1:256 in 4 and 1: 64 in 8 (Table 1). Out of the 192 Tibetan mastiffs, one was seropositive in breeding farms (1.08%) and 19 in the field (19.19%) (Table 1). As shown in Table 3, the investigation revealed that the prevalence in female animals was 7.86%, and 17.31% in male animals, but the difference was not statistically significant (*P* >0.05).

**Table 3:** **Prevalence of antibodies to *T.gondii * in Tibetan mastiffs by gender in Qinghai, China**

**Gender**	**NO. examined**	**NO. positive**	**Prevalence (%)**
Male	52	9	17.31
Female	140	11	7.86
Total	192	20	10.42

A total of 133 serum samples of yaks were seropositive (8.30%). The four counties in Qinghai Province which were tested were all infected by *T.gondii * (Table 2). The seroprevalence in male and female yaks was 7.99% and 8.57% (Table 2), but the difference was not statistically significant (*P* >0.05). As shown in Table 4, the investigation revealed the antibody titers of *T.gondii * infection in yaks and the prevalence in the age group of ≤1 year was 12.71%, 5.51% in 1–3 years, 9.11% in 4–5 years, and 17.91% in >5 years, and the difference was statistically significant (*P* <0.05).

**Table 4:** **Prevalence of *T. gondii *infection in yaks by age and antibody titers in Qinghai, China**

**Age groups**	**NO. examined**	**IHA titers**					**Positive**	
		**≤1:16**	**1:64**	**1:256**	**1:1024**	**1:4096**	**NO.**	**%**
≤1 year	236	206	15	12	3	1	30	12.71
1-3 years	762	720	9	21	4	7	42	5.51
4-5 years	538	489	18	14	6	11	49	9.11
>5 years	67	55	5	2	2	3	12	17.91
**Total**	1603	1470	47	49	15	22	133	8.30

## Discussion

Nearly one third of the global human population has been reported infected by *T.gondii *, but only a small percentage of exposed people show obvious clinical symptoms [7]. By far, toxoplasmosis has been confirmed to occur in dogs [8]. Tibetan mastiffs feed on the uncooked and raw meat, which lead to high potential to be infected by *T.gondii *. Limited knowledge about the *T.gondii * infection in Tibetan mastiffs in China is available. In the present study, the seropositivity of Tibetan mastiffs’ serum samples (10.42%) was considered higher compared to the results in pet dogs in other provinces and cities of China [9-13]. Indeed, the special geographic environment may also contribute to the high seropositivity. However, the prevalence is lower than in other cities, such as 17.5% in Guangzhou [14], 12.26% in Zhengzhou [15], 10.81% in Lanzhou [16] and 13.21% in Beijing [17]. The difference in seropositivity of *T. gondii * in Tibetan mastiffs may be resulted from living environments, serologic tests used, as well as animal husbandry practices and animal welfares. Yushu is the origin of Yangtze River while Dulan and Saishen rivers cross Wulan. A large number of felid, such as Pallas cat, *Felis bieti*, may be the source of *T. gondii * infection. In this study, there was not statistically significant difference of *T.gondii * seroprevalence between female and male Tibetan mastiffs.

The present investigation showed that the overall seropositivity was 8.30% in yaks, which was lower than Qumalai, Nangqian, Zadou, Zhiduo [6] and in a study performed partial areas in Qinghai Province [5], but higher than Tianjun [6]. As we know, yaks free-graze in the pasture, living together with other wild and domesticated animals, which have more chances to ingest *T.gondii * oocysts through the fecal-oral route. Among the four investigated areas, a higher seropositivity of *T.gondii * was found in Yushu county, compared with the other three counties (*P* <0.05). The higher seroprevalence of *T.gondii * in Yushu is also possibly due to the special geographic environment as explained previously.

A higher prevalence of infection was detected in females when comparing the gender of yaks, but the difference was not statistically significant (*P* >0.05). The comparison of the ages of yaks showed higher infection in group aged >5 years old (Table 4). Because of long-term pasture with other animals, older yaks have had more opportunities to be in contact with food contaminated with oocysts. The group of yaks ≤1 year old was ranked as the second highest prevalence of *T.gondii *. The acquisition by the fetus with *T.gondii * infection during female bovine pregnancy is also considered as an important factor of contamination. So it may be an indicator of the infection rate of congenital *T.gondii * in yaks. Although attempts to isolate *T. gondii * from seropositive cattle were often unsuccessful [18], and a study showed that viable tissue cysts can remain in cattle for up to 1 191 days [19]. So the infected yaks may be an important source of infection for other animals and humans in China.

The meat of yak is mainly consumed by people and other carnivorous animals in Qinghai Province, and under certain condition, it is consumed raw or undercooked. The prevalence of antibodies to *T.gondii * may affect infection rates in other hosts in the same region, and it was primarily confirmed by epidemiological studies of seropositivity between Tibetan mastiffs and yaks in Yushu and Wulan. In the Tibet plateau, Tibetan mastiffs and yaks are pastured together in the field. Tibetan mastiffs feed on the meat of yaks. Sometimes, the aborted fetuses of these herbivores, instead of deep burial or burning down, were also the food of Tibetan mastiffs. So in this study, the seropositivity of *T.gondii * in Tibetan mastiffs and yaks in Yushu (*P* >0.05) and Wulan (*P* >0.05) has no statistically significant difference, while Tibetan mastiffs in the field were significant higher than those in the breeding farms (*P* <0.05).

## Conclusions

In conclusion, these findings demonstrated Tibetan mastiffs and yaks in Qinghai are exposed to *T.gondii * infection, and yaks posed a potential risk of human contamination with *T. gondii*. To our knowledge, this is the first seroprevalence survey of Tibetan mastiffs infected by *T. gondii * in mainland China. It is necessary to carry out measures to prevent and control *T. gondii * infection in Tibetan mastiffs and yaks.

## Competing interests

The authors declare that they have no competing interests.

## Authors’ contributions

DLZ and HY conceived and designed the study, and critically revised the manuscript. MW, YHW, QY and PM performed the experiments, analyses the data, drafted and revised the manuscript. All authors read and approved the final manuscript.
